# Cough and Its Significance
1Dr. F. J. Smith, Brit. Med. Jour., May 28, 1904.


**Published:** 1904-08-27

**Authors:** 


### COUGH AND ITS SIGNIFICANCE.[1]

Cough is a symptom of nerve irritation, usually of a branch of the vagus, but sometimes of other peripheral nerve endings. Thus the source of irritation may be in the external auditory meatus, and it is then most frequently a plug of wax. Pharyngo-tonsillar troubles are more frequent causes of cough. They are suggested by the existence of hawking with the cough, and the expectoration is in the form of little pellets often mixed with blood. A pendulous uvula, adenoids, chronic pharyngitis are examples. The coughs of the specific fevers, pertussis, and diphtheria appear largely to take origin in the same region. It must be remembered that pertussis may occur in an adult. In laryngeal and tracheal coughs there may be a gross lesion visible to the laryngoscope or the appearances may be approximately normal. Tuberculous, syphilitic, or malignant ulceration may be seen in the larynx or there may be paresis of one of the vocal cords due to pressure exercised on the recurrent nerve by an intrathoracic aneurism or other tumour. Papillomata within the larynx are another cause of cough, more especially in children. Again, with little or no obvious change the laryngeal mucous membrane may be unduly irritable, as after influenza and other debilitating diseases; excessive use of the voice causes the same thing, and probably gout is responsible in other instances. The irritation may sometimes be more deeply seated as when an abscess, aneurism, or enlarged glands press upon the trachea or bronchi. The pulmonary causes of cough are numerous, but they are for the most part readily diagnosed on physical examination of the chest; in children, more especially, it is necessary to remember that a cough may result from the presence of a foreign body in one of the bronchi. Pleurisy has a very obvious connection with cough both in its acute and chronic forms. Stomach coughs must also be recognised among the clinical possibilities.

In the differential diagnosis, age is of some assistance. Coughs in young children are usually due to bronchial conditions, tubercle, pertussis, or pneumonia, though the chance of a foreign body must not be forgotten. Respiratory distress with cough suggests acute inflammatory trouble in the chest or bronchial or emphysematous disease producing cardiac weakness; in young children inspiratory stridor may mean diphtheria with laryngeal obstruction. Another point in diagnosis is the duration of the cough. A few days only means usually some acute irritation or inflammation; persistence for months suggests some organic change; it may be, for example, a growth within the chest which even careful physical examination may not easily discover. The apparent cause of each attack may help in recognising the source of the irritation. Thus a cough, which comes on only in the recumbent posture is indicative of a relaxed uvula or of laryngeal irritation. Both laryngeal and pulmonary coughs are apt to be excited by the patient moving into a cold bedroom or getting into bed between cold sheets, but a cough which wakes the patient after he has been asleep for a time is most likely due to disease of the lungs—tubercle or a growth, for example. Cough on waking in the morning is met with in laryngeal and bronchial catarrhs. The character of the expectoration (if any), the situation of pain, and the presence or absence of hoarseness or other change in the voice are other lines of investigation which help in tracing the irritation responsible for cough in each individual case. Treatment demands in the first place removal of the cause where this is possible. Next as a general principle one may say that a dry cough should be suppressed whilst a cough with expectoration should be encouraged. In irritation in the throat topical applications have a large area of usefulness. A krameria lozenge containing one-eighth of a grain of cocaine is valuable in relaxed uvula and pharyngeal congestion. So also is a solution of carbolic acid (1 in 60) applied on a brush to the posterior pharynx and epiglottis. Laryngeal irritation from almost any cause may be checked by the use of a continuous spray apparatus, using equal parts of ipecacuanha wine, compound tincture of benzoin, and a 5-per-cent. solution of cocaine. A useful linctus, to be taken in sips and swallowed slowly, is composed of 10 minims each of solution of morphine, syrup of lemon, syrup of squill and glycerine, with 3 minims of dilute hydrocyanic acid, and water to a drachm. To assist in getting rid of abundant secretion, as in the case of a phthisical cavity or chronic bronchitis, iodide of potassium and syrup of tolu are valuable; in strong patients the reflex stimulus of the morning "cold tub" has the same effect. The lozenge of liquorice and anise of the Brompton Hospital Pharmacopœia is a useful sedative formula. Potassium iodide is less serviceable in children than in adults; in the latter belladonna in full doses acts well.

[1] Dr. F. J. Smith, Brit. Med. Jour., May 28, 1904.

